# Localization of Piezo 1 and Piezo 2 in Lateral Line System and Inner Ear of Zebrafish (*Danio rerio*)

**DOI:** 10.3390/ijms25179204

**Published:** 2024-08-24

**Authors:** Marialuisa Aragona, Kamel Mhalhel, Lidia Pansera, Giuseppe Montalbano, Maria Cristina Guerrera, Maria Levanti, Rosaria Laurà, Francesco Abbate, José A. Vega, Antonino Germanà

**Affiliations:** 1 Zebrafish Neuromorphology Lab, Department of Veterinary Sciences, University of Messina, 98168 Messina, Italy; mlaragona@unime.it (M.A.); kamel.mhalhel@unime.it (K.M.); lidia.pansera@studenti.unime.it (L.P.); gmontalbano@unime.it (G.M.); mguerrera@unime.it (M.C.G.); mblevanti@unime.it (M.L.); laurar@unime.it (R.L.); abbatef@unime.it (F.A.); 2 Departamento de Morfología y Biología Celular, Grupo SINPOS, Universidad de Oviedo, 33006 Oviedo, Spain; javega@uniovi.es; 3 Facultad de Ciencias de la Salud, Universidad Autónoma de Chile, Santiago 7500912, Chile

**Keywords:** Piezo 1, Piezo 2, zebrafish, mechanosensory organs, inner ear, later line system, comparative medicine

## Abstract

Piezo proteins have been identified as mechanosensitive ion channels involved in mechanotransduction. Several ion channel dysfunctions may be associated with diseases (including deafness and pain); thus, studying them is critical to understand their role in mechanosensitive disorders and to establish new therapeutic strategies. The current study investigated for the first time the expression patterns of Piezo proteins in zebrafish octavolateralis mechanosensory organs. Piezo 1 and 2 were immunoreactive in the sensory epithelia of the lateral line system and the inner ear. Piezo 1 (28.7 ± 1.55 cells) and Piezo 2 (28.8 ± 3.31 cells) immunopositive neuromast cells were identified based on their ultrastructural features, and their overlapping immunoreactivity to the s100p specific marker (28.6 ± 1.62 cells), as sensory cells. These findings are in favor of Piezo proteins’ potential role in sensory cell activation, while their expression on mantle cells reflects their implication in the maintenance and regeneration of the neuromast during cell turnover. In the inner ear, Piezo proteins’ colocalization with BDNF introduces their potential implication in neuronal plasticity and regenerative events, typical of zebrafish mechanosensory epithelia. Assessing these proteins in zebrafish could open up new scenarios for the roles of these important ionic membrane channels, for example in treating impairments of sensory systems.

## 1. Introduction

Sensory system disorders characterized by an impairment of the normal processing of the different types of sensory information from the environment are often associated with trauma, infections, and pathological or age-related neurodegeneration. To elucidate the origins of these disorders and identify potential therapeutic strategies and treatments, experimental animal models including zebrafish (*Danio rerio*) have been extensively used. The effectiveness of zebrafish models in understanding human diseases, namely sensory system disorders, is due to their easy maintenance, an availability of a comprehensive gene set sharing a considerable homology with that of humans, and the impressive plasticity of their sensory epithelia [1,2,3,4,5,6,7,8]. Indeed, zebrafish have been established as a model to investigate mechanosensory systems, their development, and regeneration [9,10,11,12], as well as to assess hearing, deafness [13], and the ototoxicity of several compounds [14,15], in addition to the regeneration of h air cells [16], especially after the extensive accumulation of knowledge regarding the structure and function of zebrafish’s auditory system. The teleost’s auditory system is made of the lateral line system and the inner ear. Compared to those of the lateral line, cells in the zebrafish inner ear are more similar to their mammalian counterparts, nominating it as a more adapted system for hair cell function studies. Still, hair cells in both systems are homologous to mammalian ones and show similar responses to ototoxic damage [17,18,19]. Zebrafish and mammals’ inner ear share several sensory structures. The zebrafish inner ear consists of a labyrinth formed by three semi-circular canals connected to sensory organs, namely the utricle, a sacciform structure that connects in the lower part to the saccule. Adjacent to the saccule is the third otolith organ, the lagena, a unique end organ in zebrafish comparable to the cochlea, the mammalian auditory structure [20]. In contrast to zebrafish auditory hair cells’ regenerative capacity [17,21,22], cochlear hair cell death leads to minimal recovery of function or permanent hearing loss [20,23,24]. 

At the base of zebrafish hearing channel, the bone region is enlarged and shows dilations called bone ampullae. Each ampulla includes a round sensory epithelium called crista ampullaris. A thickening of sensory cells called the macula, whose kinocilia and stereocilia are attached to dense limestone formations called otoliths, is supplied to the utriculus, sacculus, and lagena [25]. It is known that the utricle is a vestibular organ; the sacculus is involved in sound reception, and the lagena assists with sacculus functions, playing an important role in both orientation and hearing [22]. Within the mammalian utricle and saccule, there are both morphological and spatial differences between hair cells known as type I and II hair cells [26,27]. Both cell types can be found within the macula and in the surrounding extrastriolar zones. Moreover, spatial heterogeneity in the zebrafish maculae has also been previously noted [20,24,28]. 

The lateral line system, made of a cephalic and a caudal portion, perceives the external environment pressure variations. It is made of channels that run in the sub-epithelial portion along the body, with epithelial corpuscles made of clusters of sensory hair cells (deep neuromasts) beside the on-surface free neuromasts. The hair cells have numerous stereo-cilia on the apical surface (towards the fluid) and about 150 kinocilia which are grouped in a central position [29,30]. Those ciliated cells have the body incorporated into the supporting cells, which form a tight junction both with the ciliated cell and between each other. The basal pole is in synaptic contact with an afferent and efferent fiber. The individual fibers project specifically to midbrain neurons, which respond specifically to the direction of motion coming from the superficial neuromasts and those arranged in the lateral line canals, dictated by neuromasts’ cilia [31]. Teleosts sense environmental stimuli thanks to the chemosensing and the mechanosensing systems, composed mainly by the lateral line system and inner ear, converting the mechanical stimulation into neuronal signals (mechanotransduction) [32,33,34,35,36,37]. Mechanosensing covers proprioception, nociception, and touch perception [38] and several physiological functions such as hearing, body balance, and the baroreceptor reflex, in which mechanosensitive proteins play a crucial role [32,39]. Piezo proteins, Piezo 1 and Piezo 2, are among those proteins. They are identified as mechanical ion channels activated by stretching and are located in the membrane cell [34,35]. In vertebrates including zebrafish, they show biophysical properties similar to mammalian dependent voltage channels [32]. Thus, they are evolutionarily preserved, reflecting their crucial role in fundamental physiological functions [34,35,40,41,42]. Indeed, homologs of Piezo genes have been identified in several species, from protozoa [35] to mammals, which have two Piezo genes, Piezo 1 (fam38A) and Piezo 2 (fam38B). Current research focuses on the functional role of Piezo genes in determining the biological processes in which they may be involved [43]. Both Piezo genes have been detected in mechanosensitive tissues (skin, kidney, colon, etc.). Piezo 1 acts as a mechanoreceptor, detects tensile forces, and determines neural and mesenchymal stem cell differentiation [44]. Piezo 2, however, is expressed in primary sensory neurons [45], associated with tactile pain [46,47], light touch [48,49,50], airway elongation [51], proprioception [52], heart rate regulation [53], and homeostasis of cartilage and bones [54,55]. Due to their physiological importance, aberrant functions of Piezo proteins caused by mutations with gain or loss of function are associated with various pathological conditions and diseases [39]. Piezo 2 mutations induce severe touch and proprioception loss [49,50,52,56] and physiological alterations in non-sensory cells, such as in cartilage [57,58], muscles [59], and red blood cells [60]. Moreover, it has been shown that Piezo channel regulation could be implicated in inflammatory conditions affecting sensory neurons’ mechanosensitivity [32,61,62,63]. Some studies have shown that Piezo proteins play a role in mechanical nociception [40,64]. 

Brain-derived neurotrophic factor (BDNF) is an important player during early human inner ear development. In particular, it is implicated in the survival of afferent sensory neurons and cochlear development [65,66,67]. In zebrafish, different studies have shown that BDNF is involved in the survival and development of inner ear hair cells [68], while in the lateral line, it regulates the migration of primordium and the maintenance of mechanoreceptor progenitor cells [69]. S100, however, is a calcium-binding protein, proved to be a specific marker of sensory cells in the neuromast epithelium, and was reported in mammals’ cochlear and vestibular epithelial cells as well as in the sacculus and utriculus and their afferent fibers [70,71,72]. Although the interest in Piezo proteins in zebrafish is increasing lately, nothing is currently known regarding their localization and their possible function in the zebrafish octavolateralis system [32,35,36,41,64,73,74,75,76]. Thus, the purpose of this study was to assess for the first time the localization and the potential role of Piezo proteins, Piezo 1 and Piezo 2, in the octavolateralis system (lateral line system and inner ear) of adult zebrafish. 

## 2. Results

### 2.1. Anti-Piezo 1, Piezo 2, BDNF, and s100p Specificity in Zebrafish 

The anti-Piezo 1, Piezo 2, s100p (s100 calcium binding protein), and BDNF antibodies are raised against peptides synthesized from human proteins. In a previous study, Aragona et al. [37] showed that the anti-Piezo 1 and anti-Piezo 2 immunogenic sequences and their respective zebrafish sequences match at 75.71% and 83.02%, respectively (for details see [37]). The high degree of identity between antibody immunogenicity and their corresponding zebrafish sequences and the results obtained from Western blot analyses suggest that commercial antibodies are effective in zebrafish.

The same authors have previously confirmed the specificity of anti-Piezo 1 and anti-Piezo 2 in zebrafish using Western blot analyses [37]. The blots of the zebrafish proteins incubated with anti-Piezo 1 (Cat. # PA5-106296) and anti-Piezo 2 (Cat. # PA5-72975) revealed bands of ~290 and ~300 KDa, respectively, corresponding to the molecular weights of the zebrafish Piezo 1 and Piezo 2 proteins (for blot details see [37]). The anti-BDNF and s100p antibodies’ specificity, however, has been previously proved in previous studies [68,71,77,78,79,80].

To identify the immunoreactive cells of neuromasts in the lateral line system of zebrafish, a morpho-topographical approach [6] was employed, based on cellular cytoarchitectural features in conjunction with transmission electron microscopy analysis.

### 2.2. Transmission Electron Microscopy

**Figure 1:**
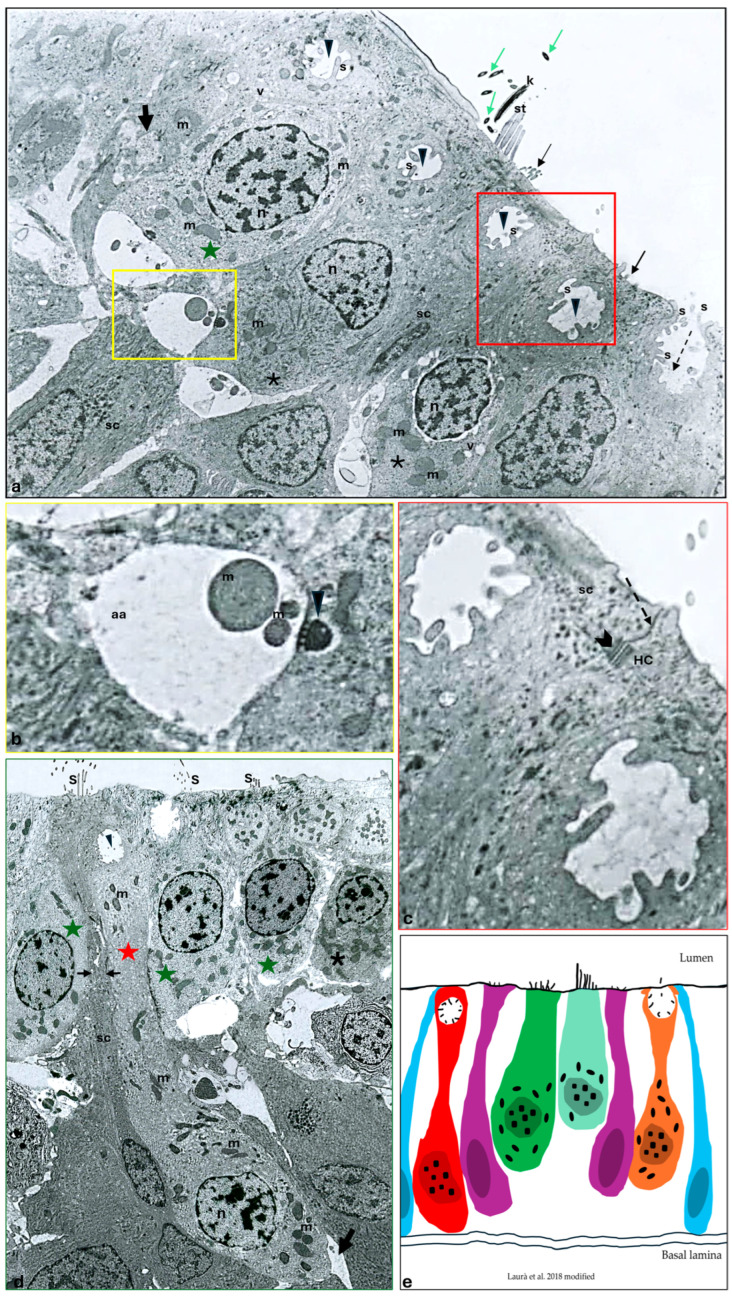
Transmission electron micrograph of a transverse section of an adult zebrafish (*D. rerio*) canal neuromast. Heterogeneity among sensory hair cells (HCs) can be observed. (**a**) A HC with a light cytoplasm (green star) and HCs with a more dense, strongly stained cytoplasm (asterisks), both with sparce heterochromatin nucleus (n), vesicle (v), and numerous electron-dense mitochondria (m), are evident. Note an HC showing a group of stereocilia with a typical staircase arrangement (st) in addition to a detached kinocilium (K) and its cross-sections (green arrows). Junction complexes between an HC and a support cell (sc) are indicated by a red inset. At the basal pole of the sensory hair cells, afferent (yellow inset) and efferent (thick arrow) synapses are visible. The occurrence of maturing HCs close to the apical surface is characterized by a peculiar crypt-like rounded space (arrowheads), with stereocilia (s). A maturing volcano-like HC, already reaching the neuromast lumen, with a peculiar depression (broken-arrow) and with the stereocilia surface projecting from the cell surface (s) to the lumen is identified. Elongated scs underneath or close to the HC, sending thin cytoplasmic projections apically, are evident. Note some microvilli (thin arrows) on the top of the sc. (**b**) Higher magnification of the basal pole of a sensory hair cell. Afferent synapse (aa) characterized by a classical pre-synaptic body (arrowhead) and a post-synaptic side with a clear cytoplasm and mitochondria (m) are visible. (**c**) Higher magnification of the apical surface of an HC and an sc. Zonula occludens at the apical surface (broken arrow) and more basally desmosomal-like junctions between the HC and sc are clearly visible (gallon arrow). Mitochondria (m). (**d**) Numerous HCs with dense (asterisk) and lighter cytoplasm (green stars) placed close to the neuromast apical surface. The occurrence of stereocilia (s) in the apical part of the HC. The occurrence of a maturing pear-shaped HC (red star) extending to the apical surface from the basal lamina to the apical surface of the neuromast. Note at the apical pole a peculiar, rounded space with some stereocilia inside (arrowhead). Note the distribution of heterochromatin in the nucleus (n). Mitochondria (m). Afferent synapse (arrow). sc with cytoplasmic projections (in between arrows) separating two adjacent HCs and extending to the apical surface. (**a**,**d**) 5000×, (**b**,**c**) 10,000×. (**e**) A graphical representation of the cell subpopulations of the neuromast sensory epithelium: mantle cells (blue), maturing crypt-like HC (red cell), sc (purple cells), hair cells with dense cytoplasm (green cell), hair cells with light cytoplasm (light green cell), maturing volcano-like HC (orange) (Laurà et al. [29] modified).

Under a transmission electron microscope, three main cell types in the *Danio rerio* canal neuromast have been observed: sensory hair cells (HCs) and non-sensory support and mantle cells. Bottle- or pear-shaped hair cells were visible in the central portion of the neuromast. Many bundles of staircase-like arranged stereocilia with a single longer kinocilium were seen on the apical surfaces of the hair cells, and a typical 9 + 2 microtubular arrangement was observed in the cross-section of the apical region of some hair cells (Figure 1a). Spherical or round central nuclei, situated slightly toward the hair cell base, were large and characterized by zones of heterochromatin spread throughout the nucleus and at the periphery. Numerous large and elongated electron-dense mitochondria were present all over the cytoplasm of the HCs (Figure 1a). In the cytoplasm, rough and smooth endoplasmic reticulum, ribosomes, Golgi apparatus, and numerous vesicles were also reported. The density of the HCs’ cytoplasm varied; some exhibited a light cytoplasm while others showed a denser—and thus, darker—cytoplasm. (Figure 1a,d). At their base, evident afferent and efferent fibers forming synapses were observed. Particularly, the afferent synapse exhibited the characteristic aspects of a ribbon synapse, with a typical presynaptic round dense body or ribbon surrounded by a ring of microvesicles and a post-synaptic side with a clear cytoplasm rich in mitochondria (Figure 1a,b). Elongated maturing HCs were also recorded. Some of them were located superficially (Figure 1a), while others extended between the cells from the basal lamina to the surface of the neuromast and were typically pear-shaped (Figure 1d). Other than the other HCs’ ultrastructural features, they showed a peculiar, crypt-like, rounded space at their apical pole, inside which some stereocilia were identified (Figure 1a,c,d). Moreover, other maturing HCs that had reached the lumen with an open crypt towards the neuromast lumen were also noted. They exhibited a peculiar volcano-like depression, into which stereocilia projected from the cell surface to the lumen (Figure 1a,d). The support cells were found underneath and close to the HCs. Moreover, cytoplasmic projections of the support cells extending to the apical surface and separating adjacent HCs were also seen (Figure 1a,d). The support cells appeared elongated, with an evident and stretched nucleus. The cytoplasm of the support cells, which had developed a rough endoplasmic reticulum and Golgi apparatus, appeared more electron-dense than that of HCs. Finally, they exhibited some microvilli at the apex (Figure 1a,d). Between these cells and the HCs’ junction systems, a zonula occludens and more basally desmosomal-like junctions were visible (Figure 1a,c). The mantle cells surrounded the HCs, forming a ring of cells enclosing the entire neuromast. The mantle cells appeared particularly thin and elongated.

### 2.3. Immunohistochemistry

The immunohistochemical analysis was carried out in serial sections employing peroxidase and double immunofluorescence labeling where s100p served as a specific marker for neuronal subpopulations of mechanosensory organs and BDNFs typically immunostaining zebrafish mechanosensory epithelia. 

#### 2.3.1. Piezo 1 and Piezo 2 Immunolocalization in the Zebrafish (*D. rerio*) Lateral Line System

**Figure 2:**
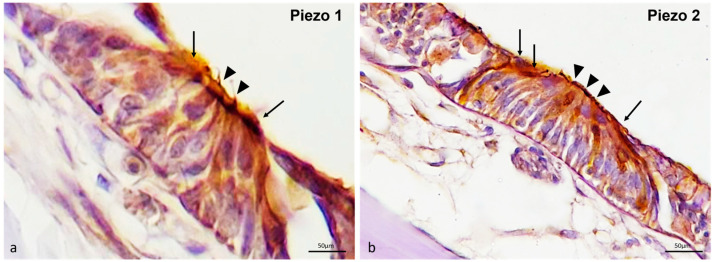
Piezo 1 and Piezo 2 immunoreactivity in the free neuromasts of the zebrafish (*D. rerio*) lateral line system. (**a**) Sensory hair cells (arrowheads) and mantle cells (arrows); Piezo 1 immunolabeled. (**b**) Sensory hair cells (arrowheads) and mantle cells (arrows); Piezo 2 immunopositive. Magnification 40×.

In the lateral line system of the zebrafish, the free neuromasts were equally immunoreactive to Piezo proteins Piezo 1 and Piezo 2. In particular, Piezo 1 (Figure 2a) and Piezo 2 (Figure 2b) were immunolocalized in the sensory hair cells and the mantle cells of the neuromasts. 

**Figure 3:**
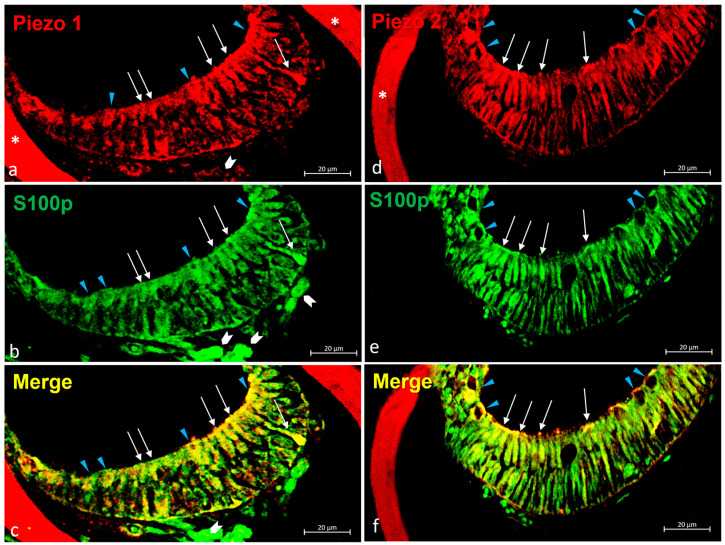
Piezo 1, Piezo 2 immunoreactivity in the canal neuromasts of the zebrafish (*D. rerio*) lateral line system. (**a**) Piezo 1 immunoreaction in sensory hair sensory cells (arrows), maturing sensory hair cells (blue arrow heads), nerve (gallon arrow), and bone (asterisk) was observed; (**b**) hair sensory cells (arrows), maturing sensory hair cells (blue arrow heads), nerve (gallon arrow) s100 immunopositive; (**c**) Piezo 1/s100p double-stained in sensory hair cells (arrows) and maturing sensory hair cells (blue arrow heads) was observed. Moreover, the nerve (gallon arrow) showed a lower double immunoreaction; (**d**) Piezo 2 immunoreaction in sensory hair sensory cells (arrows), maturing sensory hair cells (blue arrow heads), and bone (asterisk) was observed; (**e**) sensory hair sensory cells (arrows) and maturing sensory hair cells (blue arrow heads) s100 immunoreactive; (**f**) Piezo 2/s100 double-stained in sensory hair sensory cells (arrows) and maturing sensory hair cells (blue arrow heads). Magnification 40×.

In the canal neuromasts sections, the dermal bone surrounding the canal neuromast was Piezo 1 and Piezo 2 immunopositive (Figure 3a,d). The hair sensory cells were also equally immunoreactive for Piezo 1 and Piezo 2 (Figure 3a,d). The sensory nature of these cells was confirmed by their s100p immunoreactivity (Figure 3b,e). Indeed, the merged view showed the colocalization of both Piezo proteins (1 and 2) and s100p in these cells (Figure 3c,f). The sensory hair cells Piezo 1 and Piezo 2, immunolabelled, showed different architectures. Based on their morphological features and ultrastructure investigation, some of these Piezo 1 and Piezo 2 immunolabeled cells (see arrowhead in Figure 2) were identified as maturing hair sensory cells.

#### 2.3.2. Piezo 1 and Piezo 2 Immunolocalization in the Zebrafish (*D. rerio*) Inner Ear

**Figure 4:**
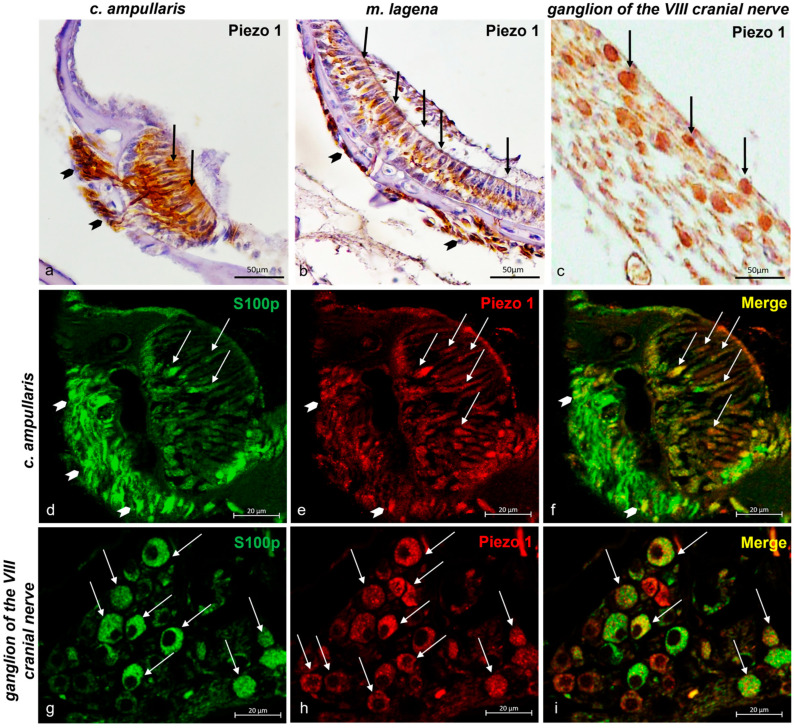
Piezo 1 immunoreactivity in the inner ear of zebrafish (*D. rerio*). (**a**) The ciliate sensory cells (arrows) in the crista ampullaris and nerve (gallon arrows) that reach the crista ampullaris Piezo 1 immunoreactive; (**b**) the ciliate sensory cells (arrows) in the macula of the lagena and nerve (gallon arrows) that reach the macula of the lagena Piezo 1 immunopositive; (**c**) ganglion of the eighth cranial nerve Piezo 1 immunostained; (**d**) sensory ciliate cells (arrows) of the crista ampullaris and nerve (arrows per gallon) that reach the crista ampullaris s100p immunoreactive; (**e**) sensory ciliate cells (arrows) of the crista ampullaris and nerve (gallon arrows) reaching the crista ampullaris Piezo 1 immunopositive; (**f**) ciliate sensory cells (arrows) of the crista ampullaris and nerve reaching the crista ampullaris (gallon arrows) Piezo 1 and s100p double-labeled; (**g**) neuron (arrows) of ganglion of the eighth cranial nerve s100p immunostained and (**h**) Piezo 1 immunoreactive; (**i**) s100p/Piezo 1 double-labeled in some neurons of the eighth cranial nerve ganglion. Magnification 40×.

In the inner ear, sensory ciliate cells both in the ampullar crest (Figure 4a) and in the macula of the lagena (Figure 4b) were immunoreactive to Piezo 1. Also, the nerves that reached the ampullar crest (Figure 4a) and the macula of the lagena (Figure 4b) were Piezo 1 immunopositive. Similarly, the ganglion of the eighth cranial nerve was Piezo 1 and Piezo 2 immunolabelled (Figure 4c). To prove the sensory cells’ nature of Piezo 1 immunoreactive cells, double labeling with the specific marker s100p antibody was conducted, in addition to a morphotopographic approach [6,68,71,79,80,81,82]. Figure 4d–f shows the sensory ciliate cells of the inner ear ampullar crest Piezo 1 and s100p immunoreactive, and some of them are s100p/Piezo 1 double-immunolabeled. Additionally, immunopositivity has been observed in the innervation reaching the crista ampullaris of the inner ear (Figure 4d–f). Similarly, the eighth cranial nerve ganglions were s100p/Piezo 1 double-labeled. Some neurons of the ganglion of the eighth cranial nerve were immunoreactive for s100p (Figure 4g), others were Piezo 1 immunopositive (Figure 4h), and only a few were s100p/Piezo 2 double-labeled (Figure 4i).

**Figure 5:**
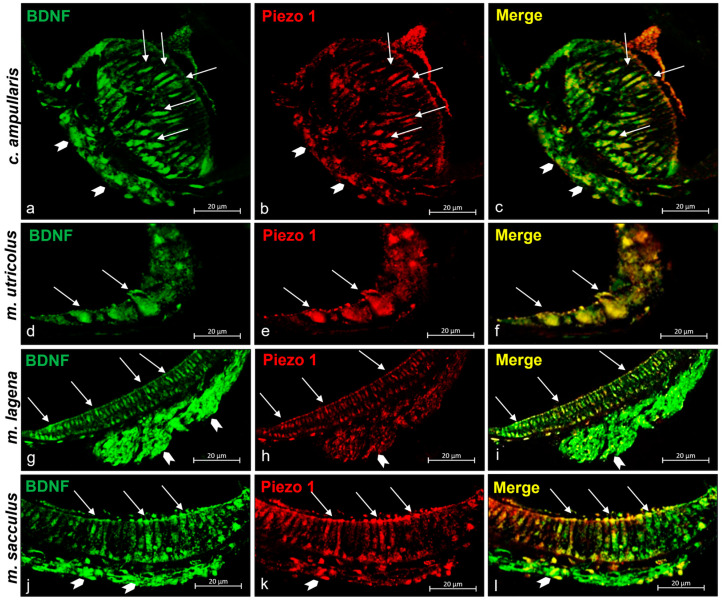
Piezo 1 immunoreactivity in the inner ear of zebrafish (*D. rerio*). (**a**) Sensory hair cells (arrows) and nerve (gallon arrows) of the ampullate crest BDNF immunoreactive; (**b**) sensory hair cells (arrows) and nerve (gallon arrows) of the crista ampullaris Piezo 1 immunopositive; (**c**) Piezo 1/BDNF double-staining in some sensory hair cells (arrows) and nerve (gallon arrows) in the crista ampullaris; (**d**) sensory cells (arrows) in the macula of the utricle BDNF immunoreactive; (**e**) sensory cells (arrows) in the macula of the utricle Piezo 1 immunopositive; (**f**) sensory cells (arrows) in the macula of the utricle BDNF/Piezo 1 double-stained; (**g**) sensory cells (arrows) and nerve (gallon arrows) in the macula of lagena BDNF immunoreactive; (**h**) sensory cells (arrows) and nerve (gallon arrows) in the macula of the lagena Piezo 1 immunostained; (**i**) sensory cells (arrows) and nerve (gallon arrows) in the macula of the lagena Piezo 1/BDNF double-labeled; (**j**) sensory cells (arrows) and nerve (gallon arrows) in the macula sacculus BDNF immunoreactive; (**k**) sensory cells (arrows) and nerve (gallon arrows) in the macula sacculus Piezo 1 immunopositive; (**l**) sensory cells (arrows) and nerve (gallon arrows) in the macula of sacculus Piezo 1/BDNF double-stained. Magnification 40×.

In addition to the localization of Piezo 1 described above, Piezo 1 immunoreactivity in the sensory cells of the macules of the sacculus and utricle as well as in the nerves reaching these structures has been observed (Figure 5d,j). Considering the already known expression of BDNF [68] in the ampullar crest cells (sensory ciliate cells, support cells) and sensory cells of the utricle, lagena, and sacculus macules, as well as the nerves reaching these inner ear structures, we are interested in evaluating the colocalization of BDNF (as shown in Figure 5a,d,g,j) and the Piezo 1 protein in cells of interest. The Piezo 1/BDNF double-staining of some crista ampullaris’ sensory cells, the macules of the lagena, sacculus, and utriculus, and the nerve that reaches these structures was recorded (Figure 5c,f,j,l). 

**Figure 6:**
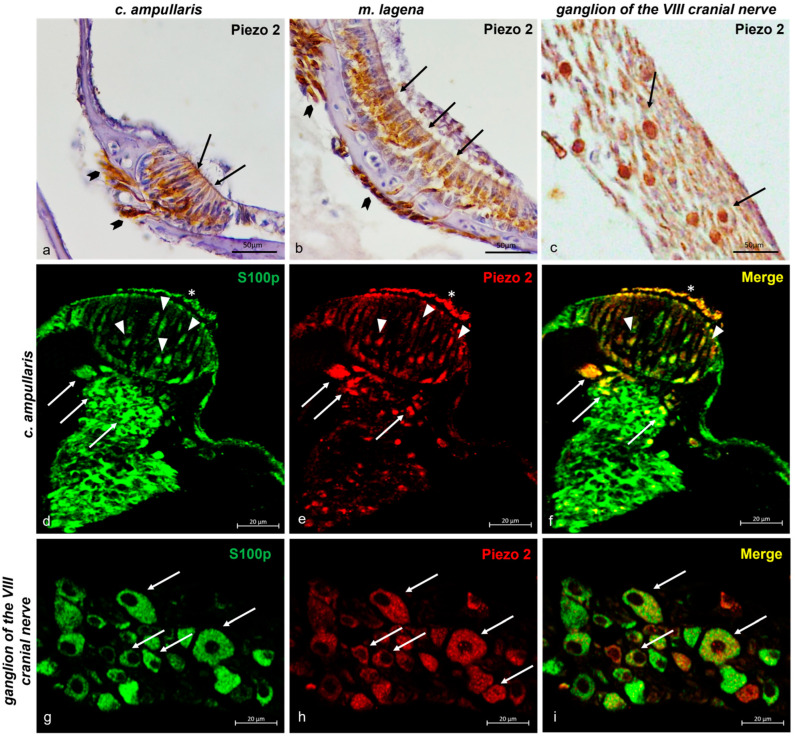
Piezo 2 immunoreactivity in the zebrafish (*D. rerio*) inner ear and ganglion of the eighth cranial nerve. (**a**) The ciliate sensory cells (arrows) in the crista ampullaris and nerve (gallon arrows) that reach the crista ampullaris Piezo 2 immunoreactive; (**b**) the ciliate sensory cells (arrows) in the macula of the lagena and nerve (gallon arrows) that reach this macula Piezo 2 immunopositive; (**c**) ganglion of the eighth cranial nerve Piezo 2 immunostained; (**d**) sensory ciliate cells (arrowheads) of the crista ampullaris, cilia (asterisk), and nerve (arrows) reaching the crista ampullaris immunoreactive to s100p; (**e**) sensory ciliate cells (arrowheads) of the crystal ampullaris, cilia (asterisk), and nerve (arrows) reaching the crista ampullaris Piezo 2 immunopositive; (**f**) ciliate sensory cells (arrowheads) of crista ampullaris, cilia (asterisk), and nerve reaching the crystal ampullaris (arrows) Piezo 2 and s100p double-labeled; (**g**) neuron (arrows) of ganglion of the eighth cranial nerve s100p (**h**) and Piezo 2 immunostained; (**i**) s100p/Piezo 2 double-labeled in some neurons of the eighth cranial nerve ganglion. Magnification 40×.

The immunoreactivity of Piezo 2 in the inner ear overlapped that of Piezo 1. Indeed, Piezo 2 was immunolocalized in the sensory ciliate cells of the crista ampullaris (Figure 6a) and in the macula of the lagena (Figure 6b), as well as in the nerve (Figure 6a,b). Moreover, Piezo 2 immunoreactivity was reported in the ganglion of the eighth cranial nerve (Figure 5c). The sensory nature of the Piezo 2 hair cells was confirmed by the double-staining with s100p (Figure 6d–f). Authors have assessed the s100p/Piezo 2 colocalization in the ganglion of the eighth cranial nerve. Some of its neurons were immunoreactive to s100p (Figure 6g), to Piezo 2 (h), and a few of them were s100p/Piezo 2 double-labeled (Figure 6i).

**Figure 7:**
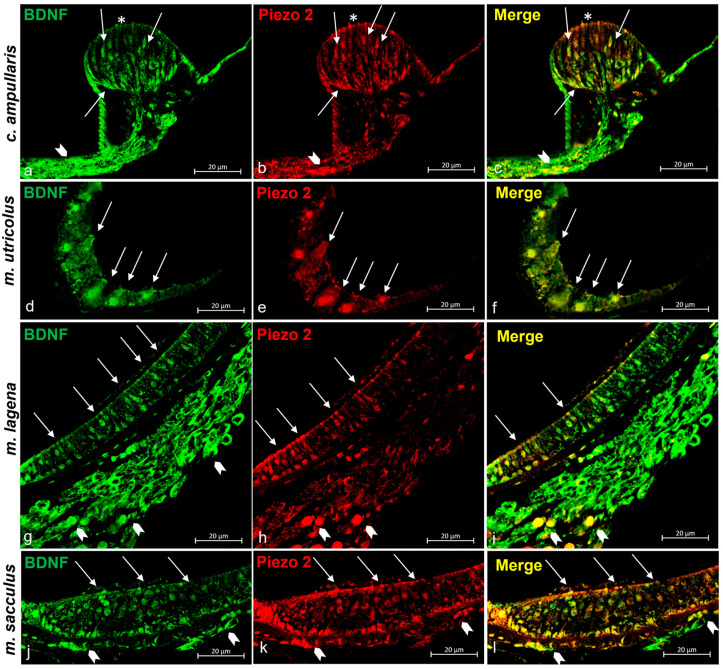
Piezo 2 immunoreactivity in the zebrafish (*D. rerio*) inner ear. (**a**) Sensory hair cells (arrows), cilia (asterisk), and nerve (gallon arrow) that reach the ampullate crest BDNF immunoreactive; (**b**) sensory ciliate cells (arrows), cilia (asterisk), and nerve (gallon arrow) reaching the Piezo 2 immunopositive ampullate crest; (**c**) Piezo 1/BDNF double-staining in some sensory hair cells (arrows), cilia (asterisk), and nerve (gallon arrow) that reaches the ampullate crest; (**d**) sensory cells (arrows) in the macula of the utricle BDNF immunoreactive; (**e**) sensory cells (arrows) in the macula of the utricle Piezo 2 immunopositive; (**f**) sensory cells (arrows) in the macula of the utricle BDNF/Piezo 2 double-stained; (**g**) sensory cells (arrows) and nerve (gallon arrows) in the macula of the lagena BDNF immunoreactive; (**h**) sensory cells (arrows) and nerve (gallon arrows) in the macula Piezo 2 immunostained; (**i**) sensory cells (arrows) and nerve (gallon arrows) in the macula of the lagena Piezo 1/BDNF double-labeled; (**j**) sensory cells (arrows) and nerve (gallon arrows) in the macula of the sacculus immunoreactive to BDNF; (**k**) sensory cells (arrows) and nerve (gallon arrows) in the macula of the sacculus immunopositive to Piezo 2; (**l**) sensory cells (arrows) and nerve (gallon arrows) in the macula of the sacculus Piezo 2/BDNF double-stained. Magnification 40×.

In addition to the ampullar crest and macula of the lagena, Piezo 2 immunoreactivity was reported as well in the sensory cells of the macules of the sacculus and utricle as well as in the nerves (Figure 7d,j). Similarly to Piezo 1, Piezo 2 has shown a colocalization with BDNF, highlighting its possible role in cell development and maintenance. The BDNFs immunoreactive in inner ear sensory epithelia are shown in Figure 7a,d,g,j, and the Piezo 2/BDNF double-stained in some sensory cells of the crista ampullaris, the lagena, sacculus, utriculus maculae, and in the nerve is shown in Figure 7c,f,j,l.

### 2.4. Statistical Analysis and Cell Counting

**Figure 8:**
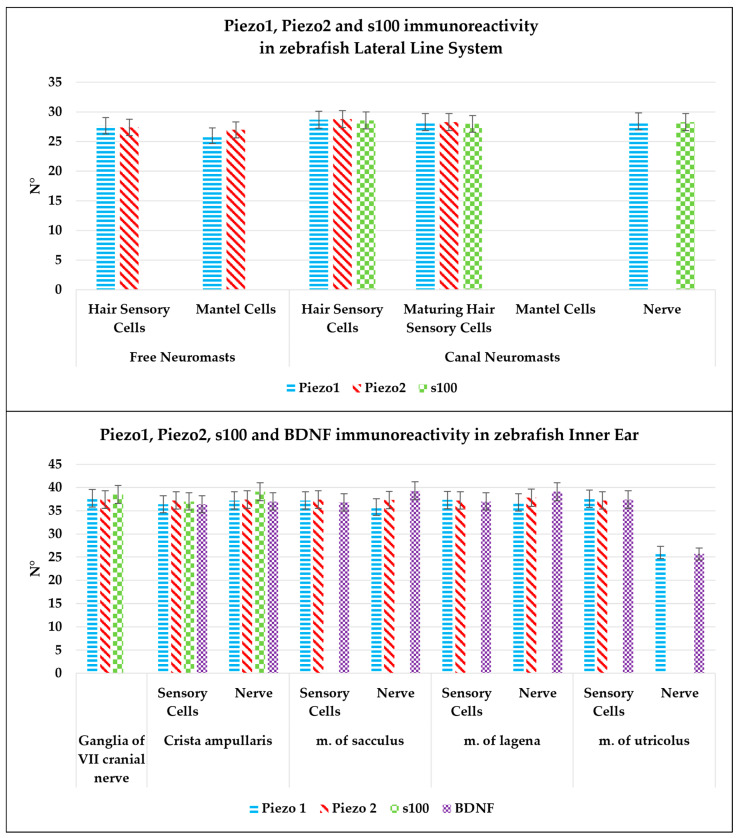
Graphical representation of immunoreactive cell counts: hair sensory cells and maturing hair sensory cells, mantle cells, and nerve in the neuromast epithelium labeled by Piezo 1, Piezo 2, and s100p; sensory hair cells and nerve of inner ear crista ampullaris and maculae epithelium immunolabeled by Piezo 1, Piezo 2, BDNF, and s100p; neurons of VIII cranial nerve immunostained with Piezo 1, Piezo 2, and s100p. The statistical analysis shows a different expression pattern of the investigated protein in different cellular subpopulations. N°: mean of cells immunopositive to Piezo 1, Piezo 2, BDNF, and s100p. The significant difference was assessed for *p* < 0.05.

According to the results of quantitative analysis, Piezo proteins (1 and 2) were immunolocalized in the lateral system and inner ear sensory epithelium and in the nerve reaching these organs. The hair sensory cells, maturing hair sensory cells, the mantle cells, and the nerve reaching the neuromast were Piezo 1 and Piezo 2 immunostained. In particular, authors have reported the expression of Piezo 1 and Piezo 2 in maturing sensory cells, while the nerve reaching the neuromast did not show Piezo 2 immunoreactivity. Finally, s100p (a specific marker of sensory cells in neuromasts epithelium) showed immunoreactivity in hair sensory cells and maturing hair sensory cells as in nerves reaching the neuromasts. Concerning the eighth cranial nerve system, the ganglions were Piezo 1 and Piezo 2 immunostained similarly to the inner ear. The sensory hair cells of crista ampullaris and the lagena, sacculus, and utriculus maculae showed Piezo 1 and Piezo 2 immunoreactivity. The comparison of the subpopulations expressing Piezo 1, Piezo 2, BDNF, and s100p cell counts in zebrafish neuromast, crista ampullaris, and maculae of inner ear sensory epithelium is illustrated in Figure 8, Table 1 and Table 2. While there were slight differences among the different subpopulations’ cells count, no statistically significant variation was detected (*p* < 0.05).

## 3. Discussion

Mechanotransduction is a fundamental process that stimulates cells to convert mechanical forces, imposed externally by environmental factors or internally by the natural microenvironment of cells’ niche, into biochemical signals [49,83,84,85,86] regulating numerous physiological processes, including touch, pain, and proprioception [39,84,87,88,89]. Although mechanotransduction triggers many biological processes, such as embryonic development, tissue repair, and regeneration, excessive and prolonged mechanical stimulation can lead to pathological processes. Despite the strong associations between mechanical signals and normal tissue homeostasis, no effective therapies are currently available for disorders related to mechanical signals [39,90]. For decades, animal models have been used to enable us to better understand the sensory pathways and the receptors affected in human diseases. Various animal models have been utilized over the years, with the zebrafish consistently regarded as one of the most effective models, among other teleosts [91], considering its various features and applications [92,93]. It was used for assessing mechanosensory systems development [94], sensorineural hearing loss resulting from damage to the sensory ciliate cells of the inner ear [6,30,95,96] and lateral line system [20,93,97], and sensitivity loss and chronic pain [98,99,100,101,102,103,104]. Moreover, diverse studies have investigated the evolution of the sensory systems and their implicated factors among the different spaces [8,105]. Indeed, they have proved that the sensory cells of the inner ear of mammals, including humans, exhibit morphological and genetic similarities with the inner ear hair cells of zebrafish. Mechanoproteins are integral to the function of sensory pathways. Dysfunctions or mutations in these proteins can lead to a variety of disorders affecting sensory perception. Piezo 1 and Piezo 2 are among this class of protein. They are essential for performing multiple physiological functions [34,35,41,75,106]. Since the discovery of their role in the detection of mechanical stress by Ardem Patapoutian (Nobel Prize 2021), they have become a promising pharmacological target for diseases [39,90]. Although they are gaining more and more interest, their localization and potential roles in the mechanosensory organs of adult zebrafish remain inadequately understood. In this study, the expression pattern and localization of Piezo 1 and Piezo 2 in the adult zebrafish octavolateralis system made of the inner ear and lateral line system have been assessed for the first time. The specificity of the used antibodies for zebrafish (anti-Piezo 1 # PA5-106296 and anti-Piezo 2 # PA5-72975) was proved by blasting the anti-Piezo 1 and anti-Piezo 2 immunogen sequences with their respective sequences from zebrafish, showing an identity of 75.71%, and 83.02%, respectively. This substantial identity proves the effectiveness of the aforementioned antibodies in zebrafish. These findings were substantiated by Western blot bands of ~290 and ~300 KDa corresponding to the molecular weight of zebrafish Piezo 1 and Piezo 2 proteins revealed by Aragona et al. [37]. In the current study, both Piezo 1 and Piezo 2 were immunolocalized in the free and lateral line channel neuromasts’ sensory epithelium of the adult zebrafish lateral line system where they seem to play their well-known function as light touch-activated mechanoproteins, as reported in previous studies [32,49]. Moreover, they could be implicated in the physiology of development and maintenance [75,107,108] to respond to physiological and non-physiological conditions [109], namely genetics, acoustic trauma, and chemical exposure [31,68,109,110,111,112]. In order to identify the Piezo 1 and Piezo 2 immunolabeled cell types, authors have conducted a thorough study using high-resolution imaging capabilities of transmission electron microscopy and double immunolabeling with s100p (s100 calcium binding protein) of the Piezo positive cells. Indeed, s100, a Ca^2+^ EF-hand binding protein regulating intracellular Ca^2+^ homeostasis as a trigger or activator protein, has been used as a marker for sensory cells, based on multiple studies [70,71,109]. Thus, Piezo 1 and Piezo 2 immunoreactive cells have been defined as neuromasts’ mantle cells and hair sensory cells, which have different morphologies; some of them have a maturing hair sensory cell feature, namely a crypt-like rounded space or a volcano-like open crypt toward the neuromast lumen, inside which some stereocilia have been identified [77]. These findings highlight the potential role of Piezo 1 and Piezo 2 in sensory cell activation and nervous cell differentiation. Piezo proteins’ expression on mantle cells, however, highlights their crucial role in the development, maintenance, and regeneration of the neuromast during continuous cell turnover [75,107,108,109,113]. These findings are in accordance with those of [32,75,76], where Piezo 2’s direct role in maintaining homeostasis and epithelial cell turnover in zebrafish was proved. Additionally, the bone component of the lateral line system where deep neuromasts are logged [6] showed high Piezo 1 and Piezo 2 immunoreactivity. These findings are in accordance with those reported by many studies [55,114], highlighting the role of Piezo proteins in bone cells [44,115,116,117,118]. Indeed, Piezo 1 and Piezo 2 channels’ expression in chondrocytes and their involvement in cartilage homeostasis associated with mechanotransduction has been proved [57,116,119]. Moreover, Piezo 1 seems to be implicated in intendon stretching and regulating senescence and apoptosis in response to mechanical stimuli within the cartilage. Piezo 2 mutation, however, could affect the musculoskeletal phenotype, affecting skeletal integrity [120]. This evidence suggests that Piezo proteins could be a potential therapeutic target for patients with osteoporosis and/or bone fractures [55]. In the inner ear, the other component of the octavolateralis system [6], Piezo 1 and Piezo 2 immunolabeled the ampullar crest’ sensory hair cells, as well as those of the sacculus, lagena, and utricle macula. These sensory hair cells have been identified as such considering their cytoarchitecture and immunoreactivity to s100p, which, other than in hair cells, has been reported in myelinated saccular nerve fibers of the sensory epithelium of the ampullar crest, the sacculus, lagena, and utricle macula [6,68,121,122,123,124,125,126,127]. These outcomes confirm the implications of Piezos proteins in mechanosensation. In addition, the ganglion of the eighth cranial nerve and its innervation was Piezo 1/s100p and Piezo 2/s100p double-stained, introducing a possible role for Piezo 1 and 2 in mechanical forces transduction, as suggested by previous studies [32,49,128,129]. Furthermore, Piezos could be implicated as lateral line neuromast sensory cells in axonal regeneration as previously suggested by [32,76,106,107,108]. Based on the findings of Germanà et al. [68] concerning the expression of BDNF in the inner ear of zebrafish, authors have considered investigating the colocalization of both Piezo proteins (1 and 2) with BDNF, proving their immunofluorescence overlapping. Given the already known involvement of neurotrophins, mainly BDNF, in the development [130], maintenance, and neuronal plasticity of the central and peripheral nervous system [130,131], the here-reported Piezo proteins/BDNF double-staining align with Piezo 1 and Piezo 2’s implication in neuronal plasticity and regenerative events, typical of zebrafish mechanosensory epithelia. Indeed, several mutations in Piezos have been linked to physio-pathological conditions including hearing impairment and deafness [35,39,40,132,133,134,135]. *Piezo* mutants, namely those including an AAAA substitution, have been reported as nonfunctional [108] when overexpressed in the inner ear via a constitutively active CAG promoter. Lee et al. [136] reported a degeneration of sensory hair cells in transgenic mice [136,137]. Even though both Piezo 1 and Piezo 2 are expressed by different genes from different chromosomes, with differential biophysical properties, such as ion selectivity, kinetics, and sensitivity to mechanical stimuli, the insignificant differences in the Piezos immunolabeled cell counts in both the lateral line system sensory epithelium and in that of the inner ear could be explained by Piezo 1 divergence from Piezo 2, as most lower organisms carry a single Piezo protein, whereas vertebrates have two [42]. Indeed, despite divergence, the proteins may have retained similar or complementary functions, necessitating their expression in the same cells.

Although limited studies on Piezo proteins in zebrafish have already begun, there is currently no evidence of their localization in zebrafish mechanosensory epithelia. Taken together, the presented evidence confirms the mechanosensing functions of Piezo proteins already stipulated in previous studies [32,35,36,39]. It highlights the potential non-sensorial role of Piezo proteins in the sensory organs, namely their implication in neuronal plasticity and regenerative events during the continuous cell turnover typical of mechanosensory epithelia in different pathological conditions such as hearing and sensitivity loss induced by age, infections, toxic compounds, pathologies, and/or trauma [6,68,127,138,139,140]. Moreover, Piezo proteins may also be involved in the mechanosensitive flow regulation in sensory neurons induced by inflammatory conditions [32,61,62,63]. Although the following study provides detailed localization of Piezo 1 and Piezo 2, it does not include functional or behavioral assays, such as touch-response or balance tests in zebrafish with altered Piezo expression. Moreover, the study was limited to adult zebrafish. Indeed, electrophysiological recordings from functional assays could link protein localization and function, while behavioral assays on zebrafish with altered Piezo expression could warrant an insight into the physiological roles of these proteins in sensory systems. Additionally, a future study covering the different stages of zebrafish development could highlight the temporal dynamics of Piezo expression.

Despite these limitations, the study contributes significantly to our understanding of Piezo proteins’ expression in zebrafish sensory systems and lays the groundwork for future research. Addressing these limitations in future studies could enhance the translational potential, opening up new scenarios for roles of these important ionic membrane channels, for example in treating impairments of sensory systems.

## 4. Materials and Methods

The specimens of adult zebrafish (*Danio rerio*) have been maintained using routine procedures [6,37,68,127]. All animal handling protocols were carried out in accordance with the principles outlined in the declaration of Helsinki and approved by the Italian Ministry of Health (A.M. n. 505/2023-PR). All procedures were conducted in triplicate, with six specimens used for each replicate.

### 4.1. Piezo 1, Piezo 2, BDNF, and s100p Specificity in Zebrafish (Danio rerio)

#### 4.1.1. Blast of the Antibody Immunogen Sequences with the Respective Zebrafish Proteins

The antibodies Piezo 1 and Piezo 2 (for antibodies’ details see Table 3) are declared to be raised against peptides synthesized from the respective human sequences. The use of the online software NCBI blastp (version 2.16.0) (protein–protein BLAST) for peptide alignment as described before [91,141] allowed the assessment of the degree of homology between the antibodies’ immunogens and the respective zebrafish sequences [37].

#### 4.1.2. Western Blot Analyses

Western blot analyses were performed as previously described [142] on the zebrafish head homogenates [37]. The anti-Piezo 1 and anti-Piezo 2 primary antibodies (Table 3) were used.

### 4.2. Transmission Electron Microscopy

Three samples by replicate were fixed in 2.5% glutaraldehyde in 0.1 M phosphate buffer (pH 7.4) at +4 °C, washed with 0.1 M phosphate buffer (pH 7.4), postfixed in 1% OsO4 in 0.2 M phosphate buffer (pH 7.4) at +4 °C for 1 h, dehydrated in graded ethanol, immersed in propylene oxide, and embedded in Durcupan (Sigma–Aldrich/Fluka, St. Louis, MO, USA). Ultrathin silver-golden sections were cut with a diamond knife on a Reichert Jung Ultracut E, placed on uncoated 200 mesh copper grids, contrasted with methanolic uranyl acetate and lead citrate, and photographed with a JEOL JEM-100 SX transmission electron microscope at 80 kV (JEOL USA, Inc., Peabody, MA, USA).

### 4.3. Immunohistochemistry

Three fresh specimens by replicate were fixed in 4% paraformaldehyde in phosphate-buffered saline (PBS) (AAJ19943K2, Thermo Scientific, Waltham, MA, USA) 0.1 m (pH = 7.4) for 12–18 h, dehydrated through graded ethanol series, and clarified in xylene for paraffin wax embedding. The included tissue samples were then cut into 7 µm thick serial sections and collected on gelatin-coated microscope slides. 

#### 4.3.1. Peroxidase Method

To assess the expression of Piezo 1 and Piezo 2 in the sensory patches of the mechanosensory system (lateral line system, inner ear) of adult zebrafish, serial sections were deparaffinized and rehydrated, washed in working buffer (Tris–HCl buffer (0.05 M, pH 7.5) containing 0.1% bovine serum albumin and 0.2% Triton-X 100) and incubated in 0.3% H_2_O_2_ (PBS) solution for 3 min to prevent the activity of endogenous peroxidase; then, to rinsed sections, fetal bovine serum (F7524 Sigma-Aldrich) was added for 30 min to avoid non-specific binding. Slide incubation with Piezo 1 and Piezo 2 rabbit polyclonal antibodies (for antibodies’ details see Table 3) was carried out overnight at 4 °C in a humid chamber. After incubation, they were washed in the working buffer and incubated for 1.5 h at room temperature with secondary antibody–peroxidase conjugate (for antibodies’ details see Table 3). The immunoreaction was visualized using 3–30-diaminobenzidine as a chromogen (DAB, Sigma-Aldrich, Inc., St. Louis, MO, USA, cat. #D5905). After rinsing in fresh water, the sections were stained with Hematoxylin nuclear staining (Bio-Optica Milano S.p.a, Milano, Italy cat. # 05-M06012). Finally, they were dehydrated, mounted, and examined under Leica DMRB equipped with a Leica MC 120 HD camera (Leica Application Suite LAS V4.7, Leica Microsystems GmbH, Wetzlar, Germany).

#### 4.3.2. Confocal Immunofluorescence

Some serial sections were treated as above and then incubated with primary antibodies. s100p rabbit polyclonal antibody and BDNF rabbit polyclonal antibody were used in double-label experiments with rabbit polyclonal anti-Piezo 1 and anti-Piezo 2, respectively (see Table 3). Since they were produced in the same host, primary antibodies were incubated sequentially. The s100p and BDNF antibodies were incubated for the first night at 4 °C in a humid chamber. After rinsing in a working buffer, the sections were incubated for 1 h at room temperature in a humid chamber with the unconjugated secondary antibody mouse anti-rabbit IgG (H + L) (see Table 3). Subsequently, the sections were rinsed in working buffers and incubated with the secondary fluorescent antibody anti-mouse IgG (H + L) Alexa Fluor 488 (see Table 3) at room temperature in a dark humid chamber for 1 h. The slides, after washing, were incubated with Piezo 1 and Piezo 2 antibodies overnight at 4 °C in a humid chamber, thoroughly washed in working buffer, and incubated for 1 h at room temperature in a dark, humid chamber with the secondary fluorescent antibody anti-rabbit IgG (H + L) Alexa Fluor 594 (see Table 3). Subsequently, excess antibody was thoroughly washed off, and sections were dehydrated and mounted with Fluoromount Aqueous Mounting Medium (Sigma Aldrich, USA). Finally, the slides were analyzed, and images were acquired using a Zeiss LSMDUO confocal laser scanning microscope with META module (Carl Zeiss MicroImaging GmbH, München, Germany) and LSM700 AxioObserver Zen 2011 microscope (LSM 700 Zeiss software ZEN 3.7). The built in “colocalization view” was used to highlight the expression of both antibodies’ signals to produce a “colocalization” signal, scatter plot, and fluorescent signal measurements. Each image was rapidly acquired to minimize photodegradation [143,144].

To provide negative controls, representative sections were incubated with specifically preabsorbed antisera as described above. Under these conditions, no positive immunostaining was observed). All immunohistochemistry experiments were conducted in triplicate.

### 4.4. Statistical Analysis

ImageJ software was used to evaluate microscope fields (ten) collected randomly [37,127,140]. One-way ANOVA was used to examine the statistical significance of the cells counting data of the different cell subpopulations in zebrafish mechanosensory epithelia (lateral line system and inner ear) immunolabeled with Piezo 1, Piezo 2, BDNF, and s100p. SigmaPlot version 14.0 (Systat Software, San Jose, CA, USA) was used to conduct the statistical analysis. An unpaired Z test was also performed. The information was given as mean values with standard deviations (Δσ). Values of *p* below 0.05 were considered statistically significant (*p* < 0.05). 

### 4.5. Cell Counting

Cell counts were performed using ImageJ (ImageJ, U. S. National Institutes of Health, Bethesda, Maryland, USA, https://imagej.nih.gov/ij/, accessed on 7 September 2022, version 1.53) [37]. Immunofluorescent microphotographs were scaled to μm and converted to grayscale, and artifacts were removed by adjusting the threshold. An area tool was used to select the region of interest. Cell numbers were expressed as counts/organ [37]. 

## 5. Conclusions

This study reported, for the first time, the localization of Piezo 1 and Piezo 2 in the mechanosensory organs, namely in the sensory hair cells of the inner ear and the lateral line system of the adult zebrafish (*Danio rerio*). Although Piezo proteins have always been considered mechanosensitive proteins, playing a critical role in mechanotransduction, new functions have been suggested in the current study, namely the activation, development, maintenance, and differentiation of sensory cells during the natural or induced turnover. These findings lay the foundation to clarify the potential role of Piezo proteins in sensory organ disorders by normal and induced neurogenesis. Future studies using the Piezo zebrafish transgenic model in the study of neurodegenerative disorders, infections, and lesions are needed to reveal the potential role of Piezo proteins in neurodegeneration and regeneration events.

## Figures and Tables

**Table 1:** Mean data ± standard deviation (∆σ) of immunoreactive cell counts: hair sensory cells, maturing hair sensory cells, mantle cells of neuromast epithelium, and nerve that reaches the neuromasts detected by Piezo 1, Piezo 2, BDNF, and s100p. The statistical analysis did not show a different expression pattern of the investigated protein in different cellular subpopulations (*p* < 0.05). The hyphen sign (-) indicates that no immunoreactivity was detected.

Antibodies	Free Neuromasts		Canal Neuromasts			
	Mean ± ∆σ of Hair Sensory Cells	Mean ± ∆σ of Mantel Cells	Mean ± ∆σ of Hair Sensory Cells	Mean ± ∆σ of Maturing Hair Sensory Cells	Mean ± ∆σ of Mantel Cells	Mean ± ∆σ of Nerve
Piezo 1	27.7 ± 3.22	26 ± 2.64	28.7 ± 1.55	28.3 ± 2.8	-	28.4 ± 4.12
Piezo 2	27.4 ± 3.5	27 ± 3.25	28.8 ± 3.31	28.3 ± 2.1	-	-
s100p	-	-	28.6 ± 1.62	28 ± 2.36	-	28.3 ± 3.33

**Table 2:** Mean data ± standard deviation (∆σ) of immunoreactive cell counts: sensory hair cells of inner ear crista ampullaris and maculae sensory epithelium, as well as nerve that reaches this sensory epithelium detected by Piezo 1, Piezo 2, BDNF, and s100p. The statistical analysis shows a different expression pattern of the investigated protein in different cellular subpopulations but the difference was still not statistically significant (*p* < 0.05). The hyphen sign (-) indicates that no immunoreactivity was detected.

Antibodies	Inner Ear								
	Ganglia of VIII Cranial Nerve	Crista Ampullaris		Macula of Sacculus		Macula of Lagena		Macula of Utricolus	
	Mean ± ∆σ of Neurons	Mean ± ∆σ of Sensory Hair Cells	Mean ± ∆σ of Nerve	Mean ± ∆σ of Sensory Hair Cells	Mean ± ∆σ of Nerve	Mean ± ∆σ of Sensory Hair Cells	Mean ± ∆σ of Nerve	Mean ± ∆σ of Sensory Hair Cells	Mean ± ∆σ of Nerve
Piezo 1	37.7 ± 1.8	36.4 ± 2.65	37.2 ± 2.56	37.2 ± 3.7	35.8 ± 2.43	37.3 ± 3.8	36.8 ± 2.7	37.6 ± 2.9	36.6 ± 3.07
Piezo 2	37.4 ± 2.2	37.2 ± 2.82	37.4 ± 3.58	37.4 ± 4.2	37.3 ± 3.8	37.2 ± 4.3	37.8 ± 1.8	37.2 ± 2.5	35.4 ± 4
BDNF	-	37 ± 2.56	39.1 ± 2.99	36.8 ± 4.37	39.3 ± 2.44	37 ± 3.97	39.1 ± 1.42	37.4 ± 3.13	36.7 ± 2.5
s100p	38.5 ± 4.6	36.4 ± 3.23	37 ± 3.71	-	-	-	-	-	-

**Table 3:** Antibodies used in this study.

**Primary** **Antibodies**	**Supplier**	**Catalog Number**	**Source**	**Diluition**	**Antibody ID**
Piezo 1	Invitrogen	PA5-106296	rabbit	1:100	AB_2853973
Piezo 2	Invitrogen	PA5-72975	rabbit	1:100	AB_2718829
s100p	Dako	Z0311	rabbit	Ready to use	AB_10013383
BDNF	Merck Millipore	AB1534SP	rabbit	1:100	AB_90748
**Secondary** **Antibodies**	**Supplier**	**Catalog Number**	**Source**	**Diluition**	**Antibody ID**
anti-rabbit IgG-peroxidase conjugate	Santa cruz Biothecnology	sc-2357	mouse	1:100	AB_628497
anti-Rabbit IgG (H + L) Unconjugated	Invitrogen	31213	Mouse	1:300	AB_228376
anti-mouse IgG (H + L) Alexa Fluor 488	Invitrogen	A-11001	Goat	1:300	AB_2534069
anti-Rabbit IgG (H + L) Alexa Fluor 594	Invitrogen	A-11012	Goat	1:300	AB_141359

## Data Availability

All data presented this study are available from the corresponding author upon responsible request.
